# Successful salvage of torsion testis by means of intraoperative indocyanine green fluorescence imaging

**DOI:** 10.1186/s40792-022-01476-9

**Published:** 2022-08-11

**Authors:** Yumi Shirasaki, Masumi Kawashima, Takuya Kimura, Hiroaki Yamanaka, Kousuke Hatta, Joel Branch, Yasuo Matsuda

**Affiliations:** 1 Department of Hepato-Biliary-Pancreatic Surgery, and Pediatric Surgery, Yao Tokushukai General Hospital, 1-17 Wakakusacho, Yao, Osaka 851-0011 Japan; 2 Department of General Internal Medicine, Yao Tokushukai General Hospital, 1-17 Wakakusacho, Yao, Osaka 851-0011 Japan

**Keywords:** Testicular torsion, Indocyanine green, Pediatric, ICG fluorescence imaging

## Abstract

**Background:**

Testicular torsion (TT) is common surgical emergency that requires early diagnosis and immediate intervention within 6 h since its onset to salvage the testis. However, the decision was made only by the surgeon’s experience whether it has to be resected or not. Recently, indocyanine green (ICG) has become an excellent tool to identify biliary and vascular anatomy, and assess perfusion abnormalities in tissues. In this case report, we successfully salvaged the twisted testis, since the testicular blood perfusion was confirmed by means of intraoperative ICG (IICG) fluorescence imaging.

**Case presentation:**

A 14-year-old healthy male patient presented due to acute left testicular pain. The patient was diagnosed with TT and had immediate surgery. Macroscopically, the testis had stagnant blood flow and appeared to be dark colored. After manual detorsion, the testis remained cyanotic and with macroscopically poor blood flow. ICG angiography was performed under near-infrared light by laparoscopic camera to assess the perfusion of the affected testicle. An excellent ICG signal appeared after 45 s in the testis, and decision was made to be preserved. Therefore, left orchidopexy was performed to complete the operation. The patient had a good postoperative course and was discharged the day after surgery. Six months later, the testis did not show any shrinkage, and both sides of the testis showed the same size without any consequences.

**Conclusion:**

The blood flow in the testis was visually confirmed during the IICG fluorescence method. ICG fluorescence imaging may become an effective alternative to evaluate whether a testis can be preserved following TT.

## Background

Testicular torsion (TT) is a common surgical emergency and requires early diagnosis and immediate intervention for testicular salvage. Most reports suggest 6 h as a ‘golden time’ for testicular viability. The viability of the salvaged testis after TT is increased if the patient presents early, however the testicular preservation rate decreases with the passage of time from onset [1, 2]. However, there is no clear standard for whether the testicle can be preserved; it is based on the surgeon’s judgement of the time elapsed from onset of the torsion and the macroscopic appearance at surgery.

Indocyanine green (ICG) is a dye used for fluorescent-guided surgery, and widely adapted in various surgical fields. ICG fluorescence imaging is emerging as major contributions to intraoperative decision-making during surgical procedures, including identification of the biliary, and vascular anatomy, and ensure adequate vascular supply for colectomy, nephrectomy, or find lymph nodes [3–6].

In this case report, we successfully salvaged the twisted testis, since the testicular blood perfusion was confirmed by means of intraoperative ICG fluorescence imaging.

## Case presentation

A 14-year-old healthy male patient presented with his parent at 1 pm at our pediatric department due to acute left testicular pain having suddenly started 30 min prior to presentation.

At the time of this presentation he was 161 cm tall and weighed 48 kg. On physical examination, his abdomen was soft and non-tender. There was left testicular tenderness, but no swelling or erythema. The left cremasteric reflex was absent.

**Fig. 1:**
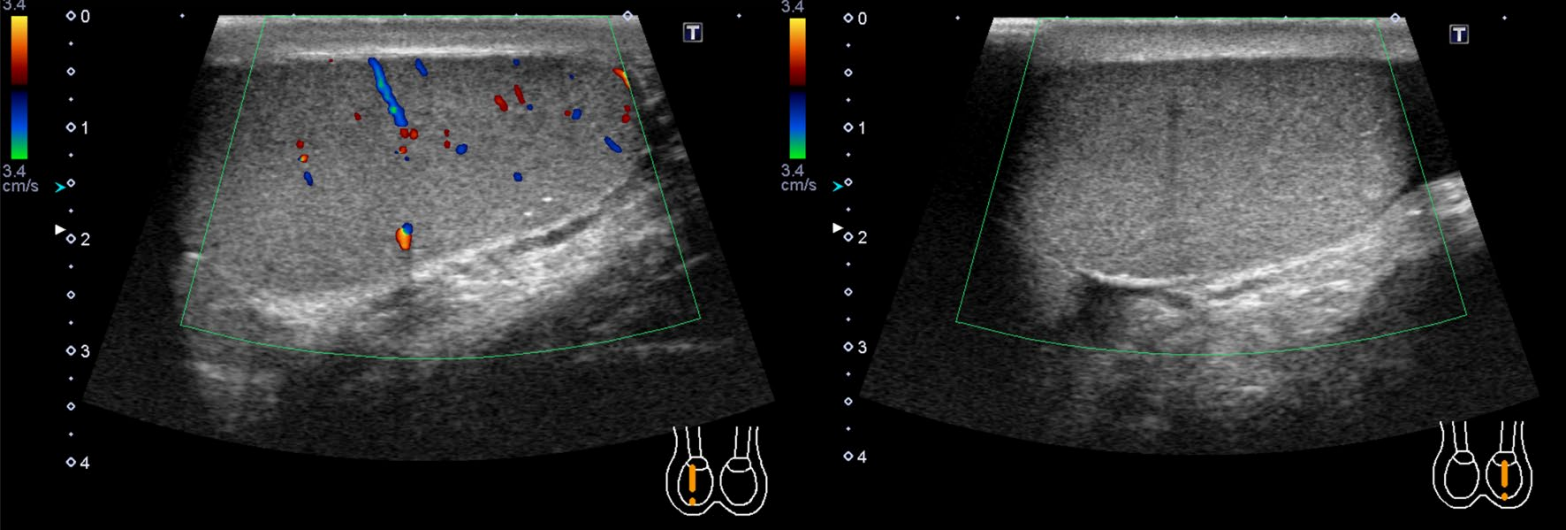
Ultrasound findings. Surface ultrasound scan of the scrotum showed absence of blood flow in the left testis. This finding was suggestive of left testicular torsion

His laboratory data revealed a mild leukocytosis (11,000/mm^3^), with other tests being normal. An ultrasound scan showed the absence of blood flow in the left testis (Fig. 1) and fluid around both testes, with a prominence on the left side.

**Fig. 2:**
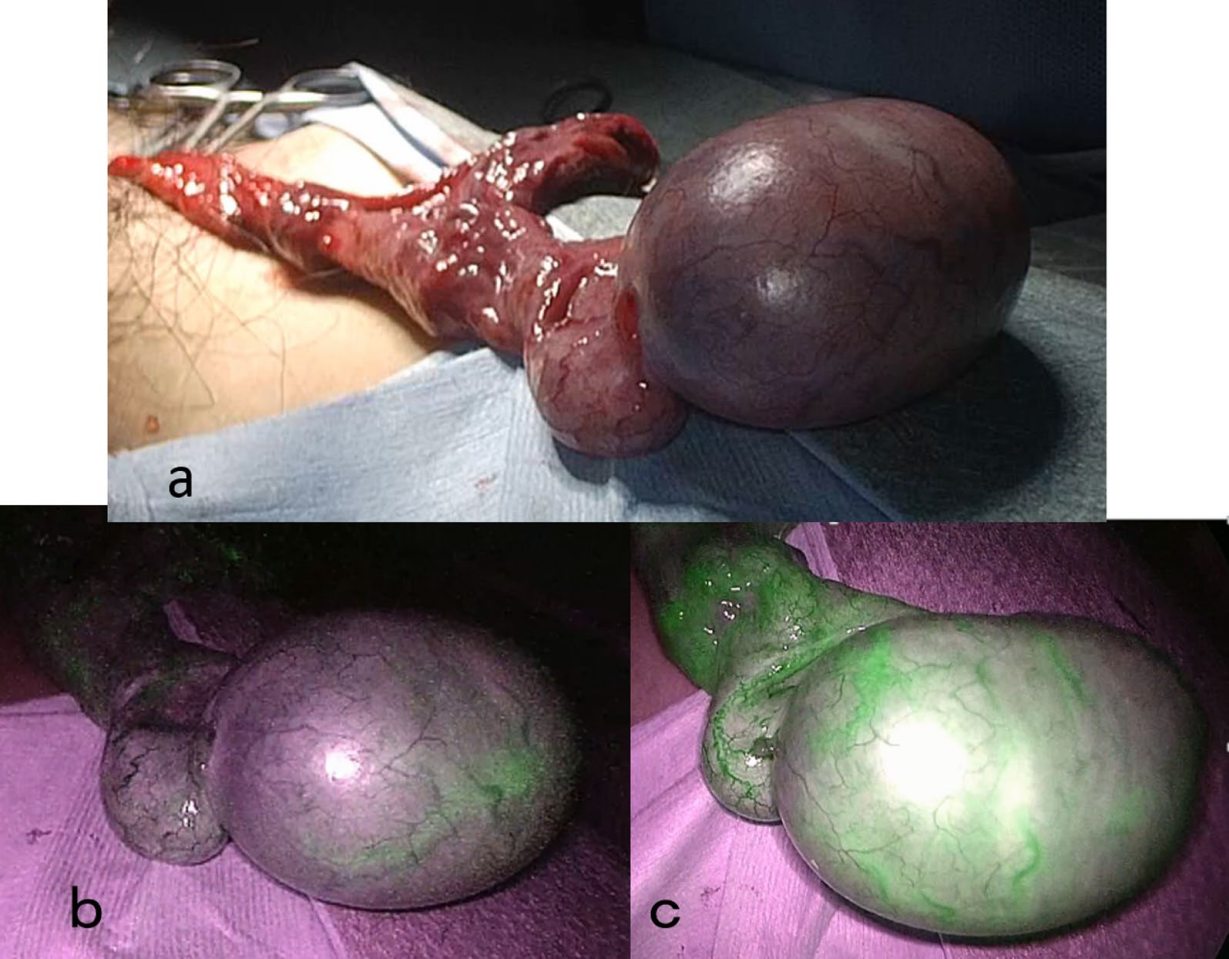
Evaluation of testicular blood flow by intraoperative ICG. **a** After manual detorsion was performed, the testis was cyanotic and showed macroscopically poor blood flow. **b** Five seconds after ICG injection, testicular vessels began to show the presence of the dye. **c** An excellent ICG dye signal appeared after 45 s and it was deemed that the testis was still viable and could be preserved

The patient was referred for immediate surgery. The time from onset of his symptoms to incision was estimated to be around 4 h. When the inguinal canal was opened from a small incision in the left inguinal region, the ischemic testis was exposed twisted and it was overtly edematous. The testis had stagnant blood flow and was dark colored. After manual detorsion was performed, the testis remained cyanotic and with macroscopically poor blood flow. The testis remained dark color and did not improve after few minutes of observation. ICG angiography was used to assess the perfusion of the affected testicle. The operating room was put into darkness to allow proper visualization of the fluorescence under near-infrared (NIR) light. The visualization of structures was performed by a laparoscope (Olympus, Tokyo, Japan) with a 30º field camera of direction, which was 10 mm in diameter and equipped with a specific filter for optimal detection of the NIR fluorescence. An excellent ICG signal appeared after 45 s from intravenous injection 2 mL (5 mL/25 mg of ICG), showing sufficient blood flow of the whole testicle (Fig. 2). Testicular blood flow was observed and it was therefore considered that the testis could be preserved. A left orchidopexy was performed to complete the operation. The patient had a good postoperative course and was discharged the day after surgery.

**Fig. 3:**
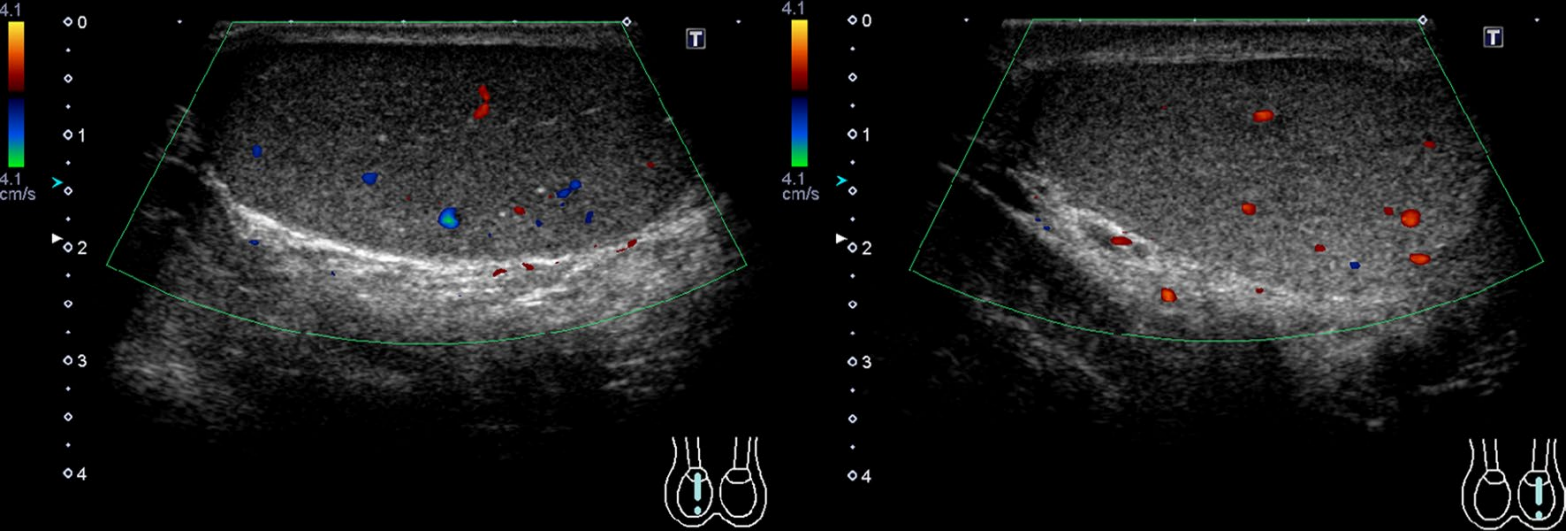
Ultrasound findings after 6 months from the operation. Six months after operation, surface ultrasound scan of the scrotum showed sufficient testicular perfusion and volume. There was no evidence of atrophy

On postoperative day 19, the patient came to our department. He experienced no particular problems except for occasional slight testicular pain. The testes showed symmetrical appearance and the location was at the bottom of the scrotum. Six months after surgery, duplex ultrasonography showed symmetrical testicular perfusion and testicular volumes (Fig. 3). The size of the left and the right testis are almost same. The left testis was 41 × 34 × 23 mm, and the right testis was 45 × 24 × 21 mm, measured by ultrasonography. The left testis was in the same volume before the operation.

## Discussion

TT occurs frequently in infancy and adolescence, and is found in 3.8/100,000 males under the age of 18 [7]. More than 30% of cases have no choice but to undergo orchidectomy. After 18 h of onset, the chance of testicular preservation is less than 50%, and after 24 h or more, the rate of testicular atrophy is close to 70% [8].

In the treatment of TT, it is important to diagnose it as soon as possible and to perform emergent surgery. At surgery, a test incision is made in the scrotum and the color tone of the testis is observed. If no problem is observed with the color tone, the testis is detorsioned and orchidopexy is performed. Even if the torsion is released, the color of the testicle may be poor, and it is often difficult to decide whether to remove or preserve it. There is also a technique that if the testicular tunica albuginea or parenchyma is incised and bleeding occurs within 10 min, it is suggestive of testicular preservation. When preserved using this technique, testicular atrophy rates have been reported to still be 17–22% [9, 10]. The criteria for determining whether a testis can be preserved or not are not clearly defined.

In this case, we evaluated testicular perfusion by means of IICG. For several years, ICG has demonstrated its application and effectiveness in tissue perfusion evaluation in a wide range of surgical procedures. In colorectal surgery, intestinal blood flow is evaluated by IICG, and it is useful for reducing the risk of anastomotic leakages [3].

In pediatric surgery, ICG is often used for example, in the identification of metastatic lesions of hepatoblastoma [4], for ICG cholangiography of biliary atresia [5], and for ICG lymphography of lymphatic malformation [6]. ICG has also been used safely and effectively in infants and newborns.

Although there is one case report of using IICG in a 26-year-old man with testicular torsion [11], there have been no reports in pediatric cases. The patient was operated after 6 and a half hours from onset of symptoms, and performed an IICG which consists of intravenous injection of 7.5 mg ICG, and visualization of the fluorescence under NIR light. ICG signals appeared in 45 s, showing homogenous vascularization of the whole testis, then the testis was salvaged. They reported to start a prospective study for ICG use in the setting of acute TT surgery in adult.

In our pediatric case, the ICG signals appeared 45 s after the intravenous of ICG injection, and we decided to preserve the testis. There are no criteria of the ICG appearance time for evaluating the blood flow of TT. Kumagai et al*.* used ICG fluorescence imaging to assess perfusion of reconstructed gastric tubes during esophageal cancer surgery [12]. After the intravenous injection of ICG, they measured the time from contrast entry into the right gastroepiploic artery base to contrast entry into the left gastroepiploic artery terminal branch and the gastric tube tip. They reported that there was a strong possibility of necrosis in the gastric tube in patients that contrast time over 90 s. In cases of non-occlusive mesenteric ischemia (NOMI), they evaluated whether ICG signals appeared or not [13, 14]. Tonooka et al. verified significance of intestinal blood flow evaluation by ICG during intracorporeal anastomosis in laparoscopic colectomy. They judged that the blood flow was good, an ICG signals appeared within 30 min [15]. Considering these reports, it seems that the testis can be preserved if ICG signals appeared at least 90 s.

The dosage of ICG in children has not been determined. Fernández-Bautista intravenously administered 0.2 mg/kg of ICG to assess the blood flow of pediatric organs [16]. There are few reports using ICG for blood flow evaluation in children, therefore more examinations are necessary to create accurate criteria for use of ICG in children.

The standard operative approach for TT is scrotal approach. In our case, an inguinal incision method was selected to evaluate blood flow from the spermatic cord to the testis. The inguinal incision method requires two incisions, the inguinal region and the bottom of the scrotum. However, in the inguinal incision method, not only the testis, but also the blood flow of the spermatic cord can be observed, which may help to determine whether or not the testis is preserved.

IICG may be a useful tool in the treatment of TT when macroscopic evaluation of the affected testis remains questionable after detorsion. In pediatric TT, whether the testis can be preserved is a very important issue for future tolerability, and it is expected that IICG evaluation will improve the testicular preservation rate.

## Conclusion

Blood flow can be successfully assessed after detorsion in adolescent patients with TT when using IICG. ICG could be a safe and an effective alternative that can be applied in pediatric cases of TT.

## Data Availability

The data that support the findings of this study are available from the corresponding author, Masumi Kawashima, upon reasonable request.

## Abbreviations

TT: Testicular torsion
ICG: Indocyanine green
IICG: Intraoperative ICG
NIR: Near infrared
